# Heterogeneity in health anxiety among medical students and HPV testing acceptability: the mediating role of eHealth literacy from a person-centered perspective

**DOI:** 10.3389/fpubh.2026.1862167

**Published:** 2026-07-06

**Authors:** Yifan Gong, Minyi Wang, Zhuo Jin, Zhihui Gu, Mengyao Li

**Affiliations:** 1 Department of Social Medicine, College of Health Management, China Medical University, Shenyang, China; 2 Beijing Aerospace General Hospital, Beijing, China

**Keywords:** eHealth literacy, health anxiety, HPV testing, latent profile analysis, medical students, protection motivation theory

## Abstract

**Background:**

Medical students, as future healthcare professionals, play a critical role in promoting preventive attitudes such as Human Papillomavirus (HPV) testing. However, their own preventive intentions may be influenced by psychological factors. This study aimed to identify latent profiles of health anxiety (HA) among medical students and examine how these profiles relate to HPV testing acceptability through the mediating role of eHealth literacy (eHL), informed by selected constructs from Protection Motivation Theory (PMT).

**Methods:**

A cross-sectional survey was conducted among 887 medical students at a university in Liaoning, China, using a voluntary response sampling method. Using the Chinese version of the Short Health Anxiety Inventory (CSHAI), the eHealth Literacy Scale (eHEALS), and a tailored acceptability scale, respectively. Data were analyzed using SPSS 25.0 and Mplus 8.3. Following descriptive and correlation analyses, Latent Profile Analysis (LPA) was employed to identify HA subgroups. To rigorously account for classification uncertainty, the Bolck-Croon-Hagenaars (BCH) method was utilized for distal outcome comparisons, followed by the construction of a simple mediation model and a covariate-adjusted multi-categorical mediation model (via a manual 3-step approach). Sequential sensitivity analyses were conducted to verify the robustness of the findings.

**Results:**

LPA identified three distinct HA profiles: “Routine” (26.2%), “Avoidance” (57.2%), and “Vigilance” (16.7%). Significant differences were observed in eHL and HPV testing acceptability across these groups (*p* < 0.001). Variable-centered analysis showed that HA was positively associated with eHL, which in turn correlated with higher acceptability. However, person-centered analysis revealed a non-linear J-shaped relationship: compared to the Routine group, the Vigilance group exhibited significantly higher eHL and testing acceptability, whereas the Avoidance group showed significantly lower levels of both. eHL was identified as a significant statistical mediator in the cross-sectional associations across these profiles.

**Conclusion:**

This study highlights the significant psychological heterogeneity of HA among medical students, identifying a complex, non-linear association between varying anxiety levels and preventive behavioral intentions. These findings may inform future educational discussions regarding the integration of digital health literacy training and psychological support among medical students.

## 1 Introduction

Cervical cancer, along with other Human Papillomavirus (HPV)-related malignancies such as head and neck cancers and anal cancer, remains a significant global health challenge, with HPV infection identified as the primary etiological factor (1–3). While HPV testing is a highly effective secondary prevention strategy, its successful implementation depends not only on public awareness but also on the proactive engagement of healthcare professionals. Medical students, as future clinicians and health promoters, play a pivotal role in counseling patients and advocating for preventive screenings (4). Their personal health beliefs and preventive attitudes are foundational to their future professional practice and the quality of care they will provide.

Health anxiety (HA) is a common psychological phenomenon among medical students, often referred to as “Medical Student Syndrome,” where exposure to medical knowledge leads to heightened concern about personal health (5, 6). Protection Motivation Theory (PMT) proposes that individuals assess health threats through threat appraisal and coping appraisal (7). However, the impact of HA on preventive intentions like HPV testing is complex. While moderate anxiety may motivate health-seeking behavior, excessive anxiety can lead to cognitive paralysis or avoidance (8, 9). Existing research often treats medical students as a homogeneous group, overlooking the diverse psychological profiles that may exist within this population (10). Identifying these heterogeneous subgroups through a person-centered approach, such as Latent Profile Analysis (LPA), is essential for developing tailored educational interventions to support students’ psychological well-being and professional development.

In the modern digitalized clinical environment, eHealth literacy (eHL), defined as the self-perceived ability to seek, find, understand, and appraise health information from electronic sources, has been recognized as an increasingly important skill for medical students in the digital healthcare environment (11–13). Higher self-perceived eHL may enhance individuals’ confidence in accessing and evaluating online health information, which could in turn influence health-related attitudes or intentions (12, 14). Within the proposed conceptual model informed by PMT, eHL may function as an informational resource that could shape how individuals respond to health-related concerns (15). Recent empirical evidence highlights the complex interplay between HA and digital health behaviors among university populations, suggesting that mitigating anxiety-related factors is intrinsically linked to improving eHL (16).

Despite the importance of these factors, few studies have explored how the interplay between distinct HA profiles and eHL influences HPV testing acceptability specifically among medical students. In this study, HPV testing acceptability refers to participants’ self-reported willingness or attitudinal openness toward undergoing HPV testing. This study aims to: (1) identify latent profiles of HA among medical students using LPA; (2) examine how these profiles differ in their acceptability of HPV testing; and (3) evaluate the mediating role of eHL in this relationship. By shifting the focus to the psychological heterogeneity of future healthcare providers, this research may offer preliminary insights for future educational discussions regarding the potential value of integrating mental health support with digital health literacy training among medical students.

## 2 Methods

### 2.1 Participants and procedures

A cross-sectional study was conducted between June and July 2024 at a medical university in Liaoning, China. Participants were recruited via a voluntary response sampling method through the Wenjuanxing online platform. To ensure data quality, the survey was distributed through official student communication channels.

The inclusion criteria were: (1) currently enrolled full-time medical students; (2) possessing basic digital literacy to complete an online assessment; and (3) voluntarily consenting to participate in this research. The exclusion criterion was the submission of invalid data, defined as surveys completed in less than 300 s or selecting the same option for all items, which suggested a lack of engagement.

Initially, 936 students engaged with the survey. After a rigorous screening process based on completion time and response consistency, 887 valid responses were retained for the final analysis, yielding an effective response rate of 94.8%. Prior to their involvement, all participants were presented with an informed consent form on the first page of the online questionnaire. This page explicitly detailed the research objectives, the principle of voluntary participation, and the strict measures taken to ensure data anonymity and confidentiality. In alignment with our voluntary response sampling method, the participants’ act of initiating the questionnaire was considered as providing explicit digital informed consent. The procedures of this study were reviewed and approved by the Ethics Committee of China Medical University.

### 2.2 Measurements

#### 2.2.1 Demographic characteristics questionnaire

A demographic questionnaire was developed for this study. Information was collected on four characteristics: gender, registered residence, number of sexual partners (in the past year), and whether participants had ever heard of HPV.

#### 2.2.2 Short health anxiety inventory

The Chinese version of the 18-item Short Health Anxiety Inventory (CSHAI) was employed to evaluate HA (17). Participants responded to each item using a 4-point Likert scale (0 = “never” to 3 = “always”). In this study, the instrument demonstrated robust construct validity, with a Kaiser-Meyer-Olkin (KMO) value of 0.945 and an explained total variance of 67.4%. The scale exhibited excellent internal reliability, evidenced by a Cronbach’s alpha coefficient of 0.938.

#### 2.2.3 eHealth literacy scale

Participants’ eHL was evaluated using the 8-item eHealth Literacy Scale (eHEALS) (18). Responses were recorded on a 5-point Likert scale (ranging from 1 = “strongly disagree” to 5 = “strongly agree”), with aggregate scores between 8 and 40. Higher totals indicate superior digital eHL. It should be noted that the eHEALS assesses self-perceived confidence in locating, evaluating, and using online health information, rather than objective digital health competence or actual behavioral capability. In the current study, exploratory factor analysis demonstrated a high KMO value of 0.947, with the single-factor structure accounting for 90.6% of the variance. The scale exhibited excellent internal reliability, evidenced by a Cronbach’s alpha coefficient of 0.985.

#### 2.2.4 HPV testing acceptability

In this study, HPV testing acceptability is defined as participants’ self-reported attitudinal willingness and openness toward undergoing HPV testing. It reflects a cognitive-attitudinal disposition. To evaluate HPV testing acceptability, a tailored four-item instrument was utilized. This instrument was developed for the present study based on a review of existing literature on HPV testing attitudes and intentions, refined through two rounds of the Delphi method. The four items assessed participants’ HPV testing acceptability. The specific item content and their corresponding factor loadings are provided in the Supplementary Tables S2, S3 and Supplementary Figure S1. Participants indicated their agreement on a 5-point Likert scale (1 = “strongly disagree” to 5 = “strongly agree”), with cumulative scores ranging from 4 to 20. Higher aggregate values indicate a more positive disposition toward HPV screening. In this study, the scale demonstrated good construct validity, as evidenced by a KMO value of 0.827 and a total explained variance of 81.4%. The scale exhibited excellent internal reliability, evidenced by a Cronbach’s alpha coefficient of 0.921.

### 2.3 Statistical analysis

Data processing and statistical evaluations were executed using SPSS (version 25.0) and Mplus (version 8.3). Initially, descriptive statistics summarized the demographic profile of the medical students, followed by Pearson’s bivariate correlation analyses to explore the interrelationships among HA, eHL, and HPV testing acceptability.

To identify heterogeneous subgroups based on psychological traits, LPA was performed in Mplus 8.3, utilizing the items of the CSHAI scale as profile indicators. We systematically compared models ranging from one to four latent profiles to determine the most parsimonious fit. Optimal model selection was adjudicated based on a comprehensive set of fit indices: lower values of the Akaike Information Criterion (AIC), Bayesian Information Criterion (BIC), and sample-size adjusted BIC (aBIC); significant *p*-values from the Lo–Mendell–Rubin (LMR) and Bootstrap Likelihood Ratio (BLRT) tests; and the requirement that each profile contains at least 5% of the total sample.

Subsequently, to rigorously account for classification uncertainty and avoid biases associated with deterministic (hard) class assignments, advanced auxiliary procedures were implemented. The robust Bolck-Croon-Hagenaars (BCH) method (19) (via Mplus 8.3) was employed to examine variations in continuous distal outcomes (HA, eHL, and HPV testing acceptability) across the identified HA profiles.

A dual-stage mediation analysis was conducted. First, a variable-centered approach utilized a simple mediation model to assess eHL as a mediator between continuous HA and testing acceptability, adjusting for prespecified covariates. Second, a more granular person-centered analysis was implemented. Specifically, a manual three-step approach (20) was employed in Mplus to execute the multi-categorical mediation model while fully maintaining the integrity of classification uncertainty. The latent HA profiles served as the independent variable, with the “Routine” group (Low-HA) designated as the reference category, while continuously controlling for covariates including gender, residence, partner status, and prior HPV awareness.

Finally, to ensure methodological rigor, a sequential sensitivity analysis was performed. We compared the indirect effect estimates of the fully adjusted multi-categorical mediation model against an unadjusted base model (without covariates) to robustly verify that the identified mediation pathways were independent of potential confounders.

## 3 Results

### 3.1 Demographic characteristics

The demographic profile of the 887 participating medical students is summarized in Table 1. The cohort was predominantly female, representing 64.7% (*n* = 574) of the total sample. 38.0% (*n* = 337) of the participants originated from rural regions. In terms of behavioral factors, a subset of the population (14.3%, *n* = 127) disclosed having at least one sexual partner within the preceding year. 96.8% (*n* = 859) of the respondents demonstrating prior knowledge of the virus (see Table 1).

**Table 1:** Descriptive statistics of medical students.

Items	Type	*n*	Percentage
Gender	Male	313	35.3%
	Female	574	64.7%
Registered Residence	Rural	337	38.0%
	Urban	550	62.0%
Number of sexual partners (in the past year)	None	760	85.7%
	One or more	127	13.0%
Have you ever heard of HPV	Yes	859	96.8%
	No	28	3.2%
Total		887	100%

### 3.2 Descriptive statistics

As shown in Table 2, the bivariate correlations among the three main variables were all positive and significant (*p* < 0.01).

**Table 2:** Correlations (*n* = 887) among the major variables.

Variable	*M*	*SD*	1	2	3
1. HA	38.988	10.634	1		
2. eHL	29.543	6.745	0.320**	1	
3. HPV testing acceptability	14.873	3.071	0.298**	0.602**	1

M, Means; SD, standard deviations; **p<0.01.

### 3.3 Latent profile analysis

LPA was conducted to identify distinct subgroups of HA. Models with one to four profiles were compared based on multiple fit indices and theoretical considerations. The optimal number of profiles was determined by evaluating several statistical and conceptual criteria. Better model fit was indicated by lower values for the AIC, BIC, and aBIC. High classification accuracy was suggested by entropy values approaching 1.0. The LMR and BLRT tests were also used, where a significant *p* value suggests superior fit over the model with one fewer profile. Finally, for a solution to be considered valid, each profile-based subgroup was required to contain at least 5% of the total sample.

**Figure 1:**
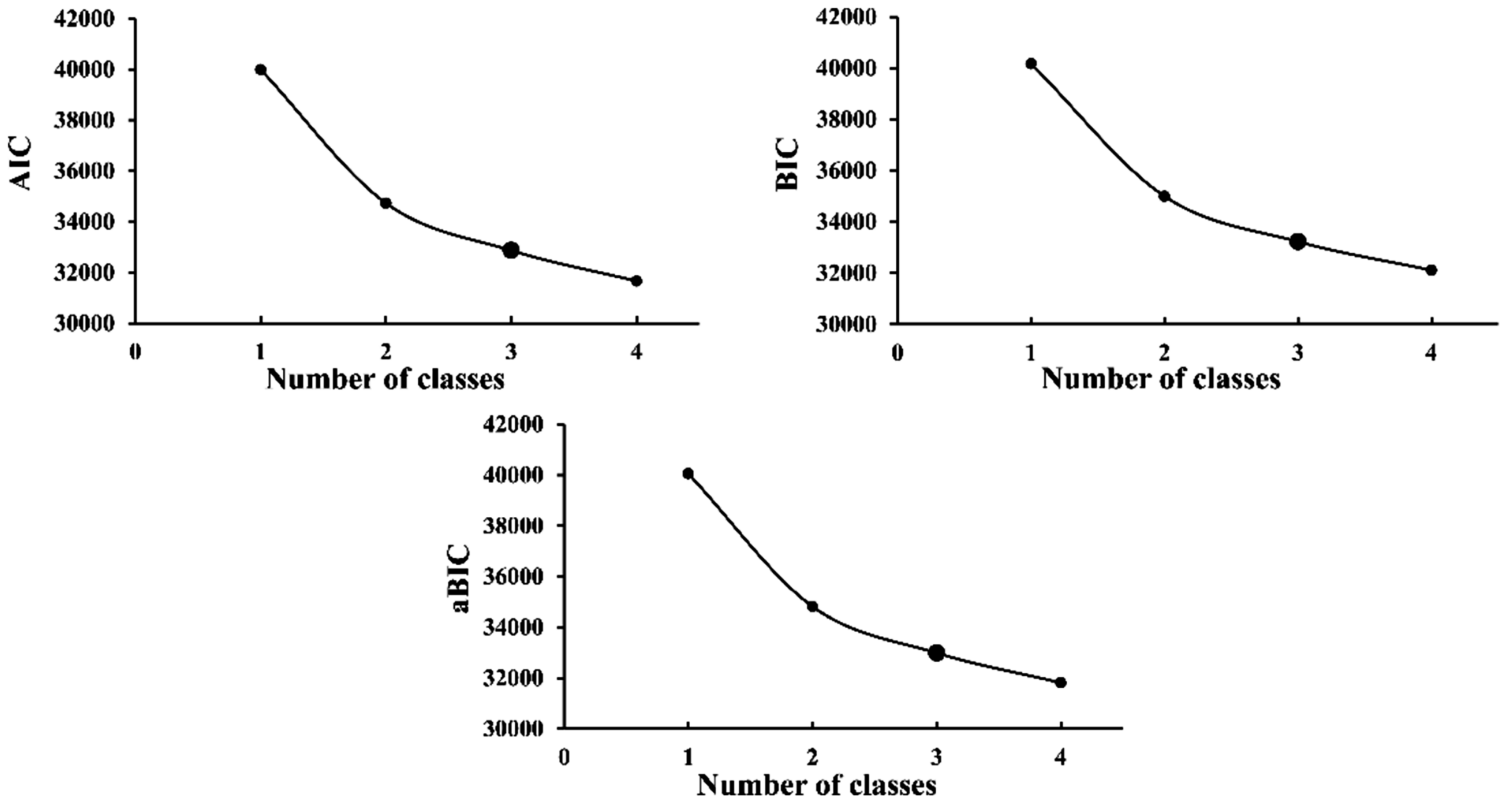
Fit indices (AIC, BIC, and aBIC) for LPA models with 1 to 4 profiles.

**Figure 2:**
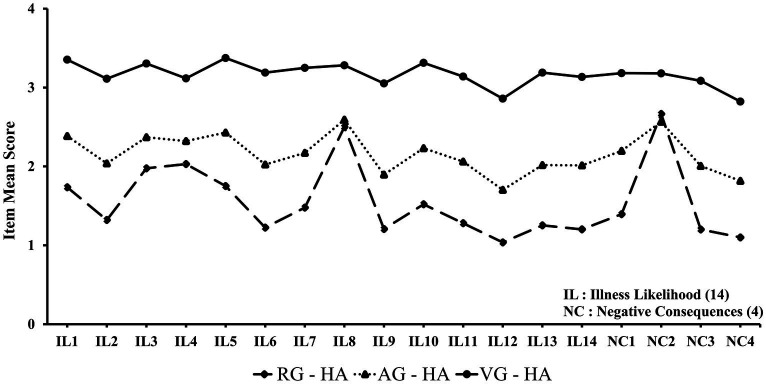
The three profiles of HA by latent profile analysis. RG, Routine Group; AG, Avoidance Group; VG, Vigilance Group.

Fit statistics are presented in Table 3. The AIC, BIC, and aBIC values decreased across models, but the reduction became less pronounced between the three- and four-profile solutions (Figure 1). Although the LMR and BLRT tests remained significant, the four-profile model yielded a subgroup with only 4.1% of participants, violating the 5% minimum size criterion. This proportion fell below the conventional 5% cutoff for a meaningful profile. Therefore, balancing model fit with parsimony, the three-profile solution was selected as the optimal model for subsequent analyses (Figure 2).

**Table 3:** Fitting index and group size of latent analysis models.

Model	AIC	BIC	aBIC	Entropy	LMR	BLRT	Group size
1C	39995.30	40167.67	40053.34	-	-	-	1
2C	34732.83	34996.16	34821.49	0.967	0.000	0.000	0.753/0.247
3C	32869.15	33223.45	32988.44	0.943	0.000	0.000	0.262/0.572/0.167
4C	31663.78	32109.05	31813.70	0.950	0.000	0.000	0.237/0.520/0.203/0.041

### 3.4 Group comparisons on eHL and HPV testing acceptability

The three identified latent profiles were conceptualized as the “Routine” group (RG-HA, 26.2%), the “Avoidance” group (AG-HA, 57.2%), and the “Vigilance” group (VG-HA, 16.7%). This nomenclature was established based on the between-group variations in HA levels and their corresponding differences (see Supplementary Table S5) in HPV testing acceptability. This classification logic is corroborated by the J-shaped association pattern identified in the restricted cubic spline (RCS) analysis (see Supplementary Figure S2). The RCS curve demonstrated that the variation in preventive willingness is not strictly linear; rather, acceptability remains at a relatively low plateau across low-to-moderate anxiety levels (encompassing the Routine and Avoidance groups) and exhibits a sharp elevation only at the high anxiety levels characteristic of the Vigilance group. It should be emphasized that these labels represent interpretive classifications informed by response patterns and theoretical considerations, rather than empirically measured behavioral states; vigilance and avoidance behaviors themselves were not directly assessed in this study.

To rigorously account for classification uncertainty and validate these patterns, the BCH procedure was utilized to compare the continuous distal outcomes across the three profiles (Table 4). Overall significant differences among the groups were found for both HA, eHL and HPV testing acceptability (*p* < 0.001).

**Table 4:** Comparisons of distal outcomes across latent profiles using the BCH procedure (*M ± SE*).

Variable	RG-HA (26.2%)	AG-HA (57.2%)	VG-HA (16.7%)	*χ^2^*	*p*
HA	27.41 ± 0.35^a^	38.95 ± 0.19^b^	57.30 ± 0.64^c^	1835.18	< 0.001
eHL	29.96 ± 0.54^b^	28.21 ± 0.27^a^	33.44 ± 0.44^c^	99.47	< 0.001
HPV Testing Acceptability	14.64 ± 0.22^a^	14.60 ± 0.13^a^	16.16 ± 0.25^b^	31.97	< 0.001

M ± SE, Mean ± Standard Error. BCH, Bolck-Croon-Hagenaars method. ^abc^ indicating significant differences between classes. *p* values for pairwise comparisons were derived from the BCH procedure.

For eHL, a distinct gradient pattern was observed: the VG-HA group reported the highest scores, the AG-HA group reported the lowest scores, and the RG-HA group scored at an intermediate level, with all pairwise comparisons being statistically significant. Notably, the lowest eHL score in the AG-HA group further supports its “Avoidance” designation, suggesting a co-occurring behavioral withdrawal from health information despite moderate anxiety.

Consistent with the J-shaped non-linear model, a different pattern emerged for HPV testing acceptability. While the highly anxious VG-HA group reported the significantly highest acceptability scores, no significant difference was observed between the RG-HA and AG-HA groups (*p* > 0.05), both groups demonstrated lower levels of acceptability.

### 3.5 Mediated effects of eHL

Two distinct analytical approaches were employed. The variable-centered approach utilized a simple mediation analysis. The person-centered approach utilized a multi-categorical mediation analysis. The mediation effect of eHL was analyzed using the Mplus 8.3.

#### 3.5.1 Variable-centered simple mediation analysis

**Figure 3:**
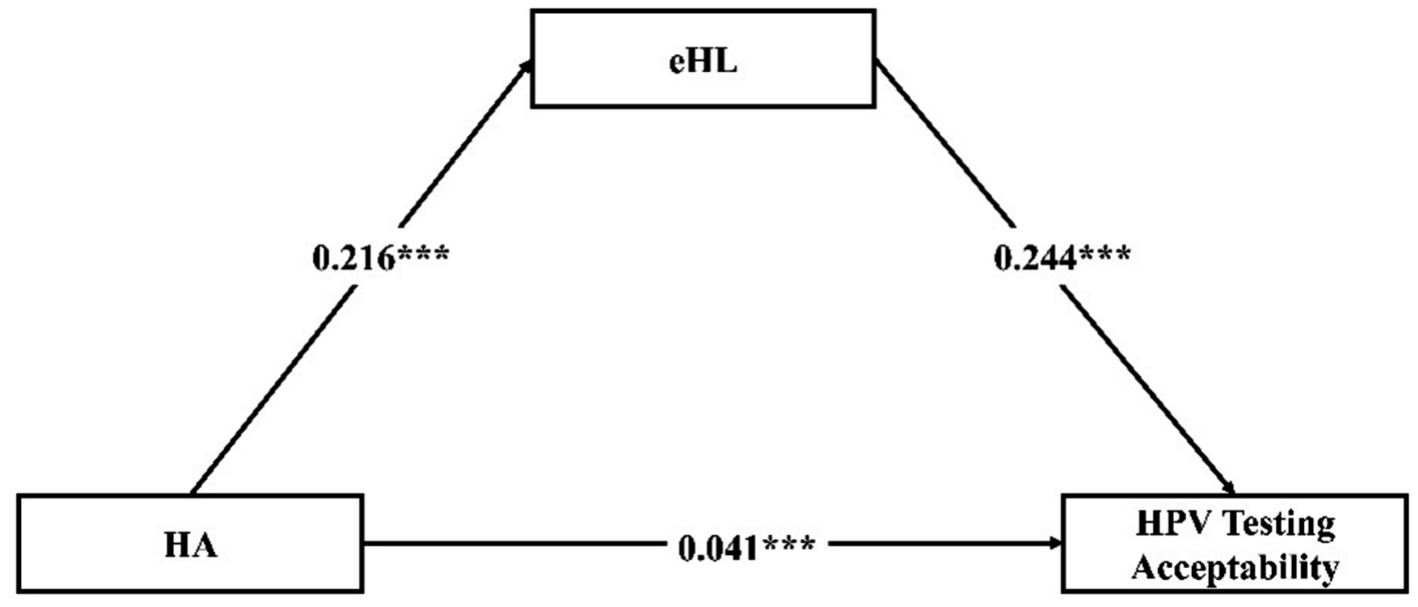
The proposed mediated model on variable level. Covariates (gender, residence, partner status, and prior HPV awareness.) were controlled in the model but omitted for visual clarity. All path coefficients shown are unstandardized. **p<0.01; ***p<0.001.

The simple mediation model was tested (Figure 3), and all pathways were found to be positive and significant after fully adjusting for covariates (gender, residence, partner status, and prior HPV awareness). HA was significantly associated with higher eHL (*B* = 0.216, *p* < 0.001), which in turn was positively associated with greater HPV testing acceptability (*B* = 0.244, *p* < 0.001). The indirect effect was statistically significant (Effect = 0.053, robust 95% CI [0.040, 0.066]). After accounting for this indirect effect, the direct relationship between HA and testing acceptability remained significant (*B* = 0.041, *p* < 0.001), indicating a partial mediation.

#### 3.5.2 Person-centered multi-categorical mediation analysis

**Figure 4:**
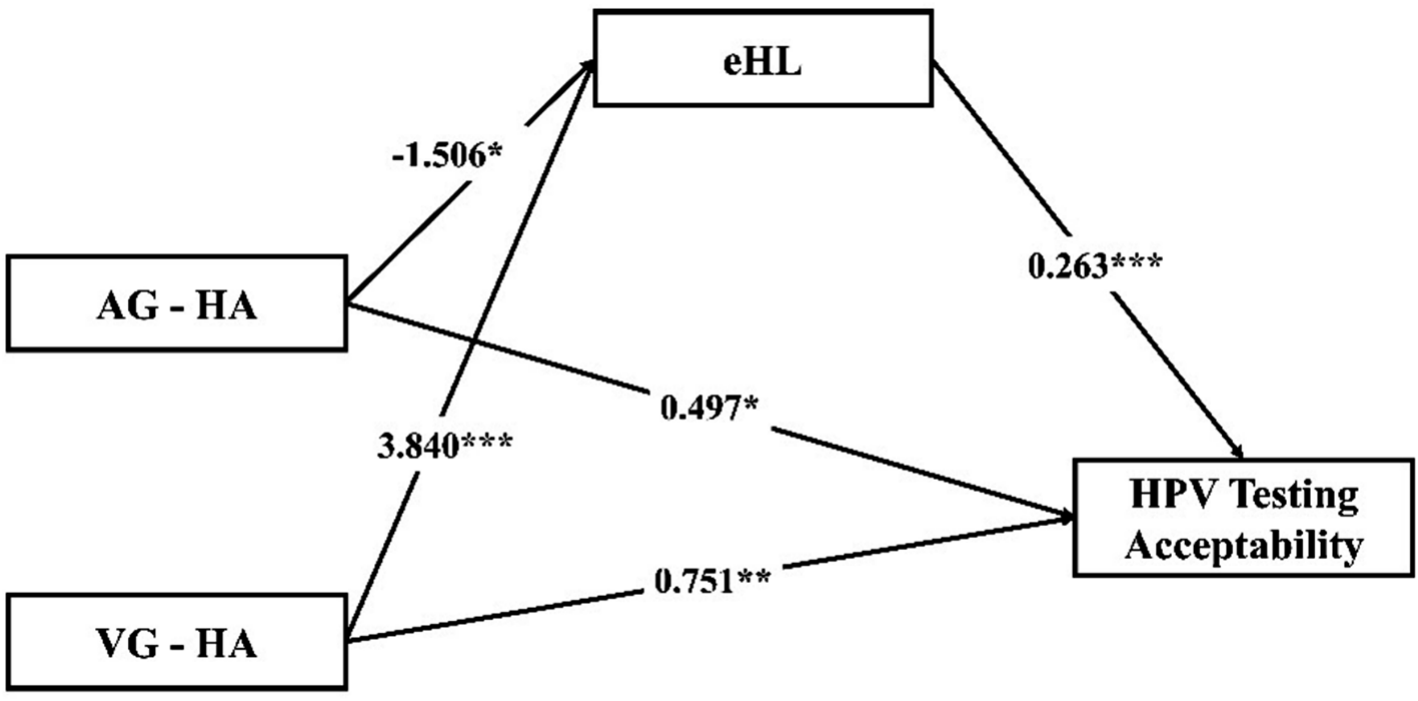
The proposed mediated model on individual level. RG, Routine Group; AG, Avoidance Group; VG, Vigilance Group, with the RG-HA serving as the reference. Covariates (gender, residence, partner status, and prior HPV awareness.) were controlled in the model but omitted for visual clarity. All path coefficients shown are unstandardized. *p<0.05; **p<0.01; ***p<0.001.

After adjusting for covariates (gender, residence, partner status, and prior HPV awareness), the multi-categorical mediation model revealed significant indirect pathways. The RG-HA profile was used as the reference group. The results are detailed below (see Figure 4).

First, the comparison between the AG-HA and RG-HA groups revealed a significant negative relative indirect effect (Effect = −0.396, robust 95% CI [−0.734, −0.058]). This was characterized by a negative association between the AG-HA profile and eHL (*a_1_* = −1.506), while eHL was positively associated with testing acceptability (*b* = 0.263). That is, compared to the RG-HA profile, the AG-HA profile was associated with a 1.506-unit lower score in eHL, and this lower eHL was statistically linked to lower acceptability of HPV testing. A significant positive relative direct effect was also found (*c’_1_* = 0.497, *p* = 0.021). However, the relative total effect was not significant (*c_1_* = 0.101, *p* > 0.05).

Next, the VG-HA group was compared to the reference group (RG-HA). A significant positive relative indirect effect was observed (Effect = 1.010, robust 95% CI [0.605, 1.414]). This pathway involved a strong positive association between the VG-HA profile and eHL (*a_2_* = 3.840). That is, the VG-HA profile was associated with a 3.840-unit higher score in eHL compared to the RG-HA profile, which was concurrently correlated with a higher level of HPV testing acceptability. The relative direct effect was also significant (*c’_2_* = 0.751, *p* = 0.008), and the relative total effect was also highly significant (*c_2_* = 1.761, *p* < 0.001). The indirect pathway accounted for approximately 57.4% of this total effect.

## 4 Discussion

This study investigated the complex relationship between HA, eHL, and HPV testing acceptability among medical students, employing a dual-methodological approach within the framework of PMT. While this study does not directly measure all PMT constructs, it draws on this framework as a conceptual lens to explore how HA (as a threat-related construct) and eHL (as a coping-related resource) may relate to preventive health intentions.

### 4.1 The heterogeneity of health anxiety in medical students

LPA identified three distinct HA profiles: “Routine,” “Avoidance,” and “Vigilance.” This foundational finding empirically supports conceptualizing HA as a set of qualitatively distinct profiles rather than a single, continuous variable. This person-centered approach aligns with current research using similar methods to uncover heterogeneity in psychological health constructs (21, 22). In the present study, 16.7% of medical students were classified into the high-HA profile. This finding aligns with literature identifying medical students as a high-risk group with a greater HA incidence than the general population (23). This phenomenon, known as “medical student syndrome” (24), is often attributed to exposure to life-threatening diseases during training, which can induce distress and maladaptive behaviors (25). In the context of medical education, such psychological distress may not only affect individual well-being but also interfere with the development of professional clinical judgment. Furthermore, factors such as female gender (26) and clinical internship experience are recognized risk factors in this population (27). However, contradictory evidence suggests HA may peak early in medical education and decrease with later clinical exposure (28). The significant differences in eHL and HPV testing acceptability observed across these profiles validated their distinctiveness and justified our person-centered analytical approach for understanding health behaviors.

### 4.2 Reconciling linear and non-linear pathways to health behavior

A key contribution of this research is its juxtaposition of variable- and person-centered analyses. The initial variable-centered analysis revealed: higher HA was associated with greater eHL, which in turn predicted higher HPV testing acceptability. This finding is consistent with the threat appraisal component of PMT, where increased Threat Appraisal (anxiety) promotes coping efforts like information seeking. However, this pathway is not universal. It contradicts a Pakistani study that reported a negative association between health literacy and HA (29). The Pakistan study involved the general public, who typically lack formal medical training and have lower overall health literacy (30). For this demographic, information seeking is often symptom-driven rather than knowledge-based (31). This situation drives them to search for health-related information online. Consequently, for them, higher eHL aids in acquiring knowledge that can mitigate HA (32).

In contrast, the sample for the present study was composed of medical students who had been exposed to at least 1 year of foundational medical education and therefore had a basic knowledge base. This pre-existing knowledge may lead to a different pattern in their professional development. Their medical knowledge may lead to a heightened awareness of disease susceptibility and specific symptoms (33). This heightened awareness can, in turn, create fear and uncertainty about their health status (34), driving them to seek electronic health information (35). For future clinicians, eHL is the ability to navigate this information to make a final health-related decision (11, 36).

However, the person-centered analysis, which accounted for latent profile heterogeneity, revealed a more intricate J-shaped relationship. Relative to the RG-HA reference group, the VG-HA exhibited significantly higher eHL, while the AG-HA displayed significantly lower eHL. This result can be interpreted through a refined application of PMT and related psychological theories:

The VG-HA Profile: The response pattern in this group is consistent with the threat appraisal component of PMT, in which higher levels of perceived threat may catalyze a constructive coping response (37). Higher HA in this profile could be linked to extensive information-seeking, which may in turn relate to their higher self-perceived eHL, although the exact directionality of this association cannot be determined due to the cross-sectional design.

The AG-HA Profile: The pattern observed in this group could potentially be interpreted in light of cognitive avoidance, although such mechanisms were not directly assessed in the present study. One possible interpretation is that anxiety in this group was sufficient to cause distress but insufficient to trigger proactive coping; under such circumstances, moderate anxiety may reflect a tendency toward cognitive avoidance (38), whereby individuals disengage from threatening information to mitigate negative affective states — a pattern that is consistent with predictions of the Vigilance-Avoidance Theory (39). This interpretation should be regarded as tentative, given that the present data demonstrate lower eHL and lower HPV testing acceptability in this group but do not establish avoidance as the underlying mechanism. If supported by future studies that directly measure avoidance behaviors, this finding could add nuance to PMT by suggesting that moderate psychological stress may suppress, rather than promote, active professional coping.

### 4.3 The central role of eHL as a coping mechanism

Across all analyses, eHL was a robust positive predictor of HPV testing acceptability. This finding is consistent with the hypothesized role of eHL as a coping-related informational resource, though the cross-sectional design precludes confirmation of temporal or causal mediation, within a PMT-informed conceptual framework. Extensive evidence shows a positive association between higher eHL and adopting preventive health behaviors (40–42).

Beyond the eHL, our findings are also consistent with the broader cancer screening literature, in which health literacy has been consistently identified as an important determinant of screening awareness, intention, navigation of screening services, and uptake (43, 44). In the specific context of cervical cancer prevention, prior work has shown that women with higher health literacy demonstrate greater understanding of HPV-related risk, more positive attitudes toward screening, and more consistent engagement with screening pathways (45). This observation is also consistent with established behavioral frameworks applied to screening contexts. For instance, the Capability-Opportunity-Motivation-Behavior (COM-B) model, which underpins the Behavior Change Wheel (BCW), positions psychological capability—including health-related knowledge and literacy—as a core upstream determinant of health behavior (46). Similarly, the Theoretical Domains Framework (TDF), which operationalizes the COM-B model into actionable domains, identifies “Knowledge” and “Skills” as discrete behavioral determinants relevant to screening engagement (47). Building on these macro-level theories, contemporary screening-specific frameworks—namely the Integrated Screening Action Model (I-SAM) (48) and the Determinants Of Screening upTake (DOST) model (49)—explicitly conceptualize health literacy, knowledge, and information-processing capacity as core upstream prerequisites for informed screening decision-making. Situating our findings within this wider body of evidence suggests that the role of eHL observed here may extend beyond digital information seeking and reflect a more general cognitive-informational capacity relevant to preventive screening decisions.

Within our PMT-informed framework, this cognitive-informational capacity directly facilitates the coping appraisal process. High eHL enhances both Response Efficacy (confidence in testing effectiveness) and Self-Efficacy (perceived ability to act). The results of this study confirm that higher eHL translates to stronger intentions for protective health behaviors, solidifying its role as a key pathway from psychological state to action. Given the digital transformation of healthcare, eHL may represent an important informational resource for medical students, potentially supporting their confidence in navigating health information for personal and future professional purposes.

## 5 Implications for theory and practice

From a practical perspective, the findings suggest that uniform approaches to health education may not equally benefit all students, and that future intervention research could explore whether profile-based strategies offer advantages (50, 51). By mapping profile-specific psychological barriers to concrete educational strategies, several translational avenues could be considered.

For students with VG-HA, future interventions might consider integrating digital information appraisal training leveraging PMT factors. This would help channel their heightened motivation by teaching them to critically evaluate online sources, thereby preventing cognitive overload or misinterpretation. For those with AG-HA, addressing underlying cognitive avoidance is critical. Introducing targeted coping-skills modules—alongside emotional regulation training and gradual information exposure—may warrant investigation before standard preventive educational content can be effective. For students with RH-HA, approaches that raise awareness and personalize risk perception could be explored to stimulate an appropriate level of health engagement.

It should be noted that these suggestions remain hypothetical and require empirical testing through prospective intervention studies.

## 6 Limitations and future directions

Several limitations must be acknowledged. First, the cross-sectional nature of this study necessitates the use of longitudinal designs in future research. Second, HPV testing acceptability was assessed as self-reported willingness rather than actual clinical uptake. Although we focused on specific psychological pathways, we did not directly measure established behavioral constructs. Future research could strengthen mechanistic insights by strictly operationalizing frameworks like the PMT and exploring moderating health attitudes (52). Third, actual testing decisions are shaped by broader unmeasured determinants, including social norms, stigma, and accessibility. Because individual literacy and behaviors are inherently embedded in organizational contexts (53), future studies should adopt multilevel designs to evaluate how school-level characteristics or medical curricula influence medical students’ eHL. Finally, the lack of sex-stratified analyses and the exceptionally high baseline HPV awareness (96.8%) limit the generalizability of our findings, highlighting the need for validation across more diverse populations.

## 7 Conclusion

This study identifies a complex, non-linear association between health anxiety and HPV testing acceptability among medical students. By employing a person-centered approach, this research revealed three distinct HA profiles (“Routine,” “Avoidance,” and “Vigilance”), which are linked to different patterns of health information-seeking and preventive intentions. Notably, the finding that moderate anxiety is associated with lower HPV testing acceptability provides an insight into potential health-related avoidance mechanisms among medical students.

Furthermore, eHL was statistically associated with the relationship between health anxiety profiles and HPV testing acceptability, suggesting a potential informational pathway that warrants further longitudinal investigation. These results suggest that eHL may serve as an important informational resource and a relevant educational target for future healthcare professionals. These findings suggest that medical education efforts could benefit from considering the psychological heterogeneity of students. Future intervention research could explore whether strategies designed to improve both digital health literacy and psychological resilience support more consistent preventive health attitudes among medical students.

## Data Availability

The raw data supporting the conclusions of this article will be made available by the authors, without undue reservation.
