# Supplementary material for: Heterogeneity in health anxiety among medical students and HPV testing acceptability: the mediating role of eHealth literacy from a person-centered perspective
Source: Front Public Health. 2026 Jul 6;14:1862167. doi: 10.3389/fpubh.2026.1862167 (PMC13381439; doi:10.3389/fpubh.2026.1862167)
Supplement: Supplementary file 1 [file Data_Sheet_1.docx]

**Supplementary Material**

Supplementary Table S1. Confirmatory factor analysis of the eHealth Literacy Scale (eHEALS)

| Item Code | Standardized Loading (*λ*) |
| --- | --- |
| Item 1 | 0.919 |
| Item 2 | 0.953 |
| Item 3 | 0.955 |
| Item 4 | 0.954 |
| Item 5 | 0.955 |
| Item 6 | 0.945 |
| Item 7 | 0.944 |
| Item 8 | 0.930 |

Supplementary Table S2. Items and confirmatory factor analysis of the HPV Testing Acceptability Scale

| Item Content | Standardized Loading (*λ*) |
| --- | --- |
| 1. Would you go to a hospital for HPV testing? | 0.798 |
| 2. Do you hope that your (future) partner will undergo HPV testing? | 0.955 |
| 3. Are you looking forward to reviewing your HPV test results? | 0.844 |
| 4. If your HPV test result is positive, would you seek treatment at a hospital? | 0.829 |


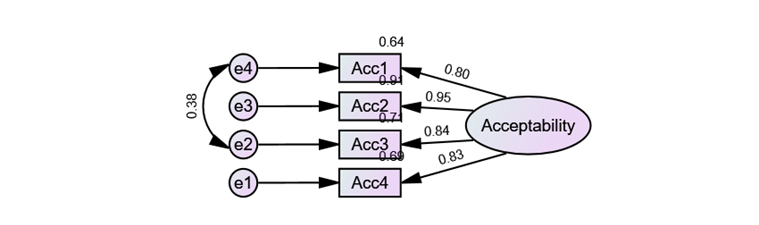


Supplementary Figure S1. Confirmatory factor analysis model of the 4-item HPV Testing Acceptability Scale.

Supplementary TableS3. Fit indices for the confirmatory factor analysis model

| Fit Index | Acceptable Value | Result | Fit Index | Acceptable Value | Result |
| --- | --- | --- | --- | --- | --- |
| *χ^2^*/*df* | < 5.0 | 10.508 | RFI | > 0.90 | 0.978 |
| SRMR | < 0.05 | 0.008 | IFI | > 0.95 | 0.997 |
| GFI | > 0.90 | 0.994 | TLI | > 0.95 | 0.98 |
| AGFI | > 0.80 | 0.941 | CFI | > 0.95 | 0.997 |
| PGFI | > 0.50 | 0.099 | RMSEA | < 0.08 | 0.104 |
| NFI | > 0.90 | 0.996 | RMR | < 0.05 | 0.006 |

*Note*. *χ^2^*/*df* = Ratio of chi-square to degrees of freedom; SRMR = Standardized Root Mean Square Residual; GFI = Goodness-of-Fit Index; AGFI = Adjusted Goodness-of-Fit Index; PGFI = Parsimonious Goodness-of-Fit Index; NFI = Normed Fit Index; RFI = Relative Fit Index; IFI = Incremental Fit Index; TLI = Tucker-Lewis Index; CFI = Comparative Fit Index; RMSEA = Root Mean Square Error of Approximation; RMR = Root Mean Square Residual.

Supplementary Table S4. Average posterior classification probabilities for the latent profiles of health anxiety.

| Latent profile | RG-HA | RG-HA | RG-HA |
| --- | --- | --- | --- |
| RG-HA | 0.967 | 0.033 | 0.000 |
| AG-HA | 0.017 | 0.976 | 0.006 |
| VG-HA | 0.000 | 0.017 | 0.983 |

Supplementary Table S5. Means and standard deviations of the 18 CSHAI items across latent profiles

| Item Code | RG-HA  (*n*=232) | AG-HA  (*n*=507) | VG-HA  (*n*=148) | Total  (*n*=887) |
| --- | --- | --- | --- | --- |
| Item 1 | 1.73 ± 0.59 | 2.39 ± 0.66 | 3.35 ± 0.61 | 2.38 ± 0.82 |
| Item 2 | 1.32 ± 0.53 | 2.04 ± 0.63 | 3.11 ± 0.72 | 2.03 ± 0.84 |
| Item 3 | 1.96 ± 0.84 | 2.38 ± 0.65 | 3.30 ± 0.58 | 2.42 ± 0.82 |
| Item 4 | 2.02 ± 1.02 | 2.33 ± 0.69 | 3.12 ± 0.72 | 2.38 ± 0.87 |
| Item 5 | 1.74 ± 0.71 | 2.43 ± 0.74 | 3.37 ± 0.64 | 2.41 ± 0.88 |
| Item 6 | 1.21 ± 0.42 | 2.03 ± 0.57 | 3.20 ± 0.71 | 2.01 ± 0.85 |
| Item 7 | 1.47 ± 0.68 | 2.17 ± 0.64 | 3.26 ± 0.64 | 2.17 ± 0.86 |
| Item 8 | 2.49 ± 1.10 | 2.60 ± 0.77 | 3.28 ± 0.71 | 2.68 ± 0.90 |
| Item 9 | 1.20 ± 0.40 | 1.90 ± 0.56 | 3.05 ± 0.75 | 1.91 ± 0.82 |
| Item 10 | 1.52 ± 0.56 | 2.23 ± 0.56 | 3.31 ± 0.59 | 2.23 ± 0.80 |
| Item 11 | 1.28 ± 0.45 | 2.06 ± 0.53 | 3.16 ± 0.74 | 2.04 ± 0.81 |
| Item 12 | 1.04 ± 0.25 | 1.70 ± 0.59 | 2.86 ± 0.91 | 1.72 ± 0.83 |
| Item 13 | 1.25 ± 0.46 | 2.02 ± 0.54 | 3.19 ± 0.69 | 2.01 ± 0.83 |
| Item 14 | 1.20 ± 0.44 | 2.01 ± 0.61 | 3.14 ± 0.71 | 1.99 ± 0.85 |
| Item 15 | 1.39 ± 0.69 | 2.20 ± 0.67 | 3.18 ± 0.71 | 2.15 ± 0.89 |
| Item 16 | 2.67 ± 1.16 | 2.56 ± 0.76 | 3.18 ± 0.66 | 2.69 ± 0.90 |
| Item 17 | 1.20 ± 0.43 | 2.00 ± 0.61 | 3.09 ± 0.71 | 1.98 ± 0.84 |
| Item 18 | 1.10 ± 0.34 | 1.81 ± 0.57 | 2.83 ± 0.91 | 1.80 ± 0.81 |


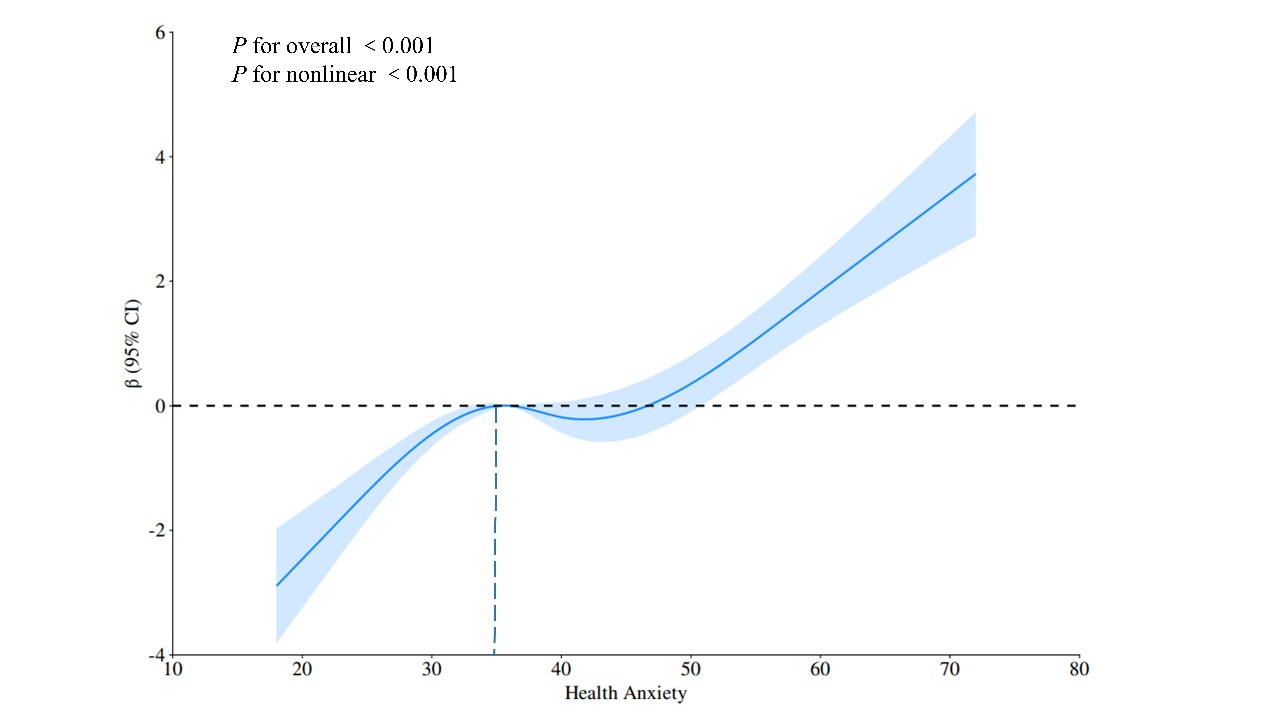


Supplementary Figure S2. Dose-response relationship between health anxiety and HPV testing acceptability based on restricted cubic spline (RCS) analysis.

Supplementary Table S6. Sequential sensitivity analyses of the multi-categorical mediation model (Unadjusted vs. Adjusted).

| Mediation Paths | Model 1 (Unadjusted) | | Model 2 (Fully Adjusted) | |
| --- | --- | --- | --- | --- |
|  | Estimate (95% CI) | *P*-value | Estimate (95% CI) | *P*-value |
| Path *b* (Mediator to Outcome) |  |  |  |  |
| eHL → HPV Testing Acceptability | 0.273 (0.241, 0.306) | < 0.001 | 0.263 (0.230, 0.296) | < 0.001 |
| AG-HA vs. RG-HA |  |  |  |  |
| Relative Path *a_1_* | -1.815 (-3.104, -0.526) | 0.006 | -1.506 (-2.789, -0.224) | 0.021 |
| Relative Indirect Effect (*ab_1_*) | -0.496 (-0.850, -0.142) | 0.006 | -0.396 (-0.734, -0.058) | 0.022 |
| Relative Direct Effect (*c*’*_1_*) | 0.445 (0.016, 0.873) | 0.042 | 0.497 (0.075, 0.918) | 0.021 |
| VG-HA vs. RG-HA |  |  |  |  |
| Relative Path *a_2_* | 3.381 (1.986, 4.776) | < 0.001 | 3.840 (2.417, 5.264) | < 0.001 |
| Relative Indirect Effect (*ab_2_*) | 0.924 (0.521, 1.328) | < 0.001 | 1.010 (0.605, 1.414) | < 0.001 |
| Relative Direct Effect (*c*’*_2_*) | 0.567 (0.018, 1.117) | 0.043 | 0.751 (0.193, 1.308) | 0.008 |

*Note:* Model 1 represents the base model without covariates. Model 2 controls for gender, residence, partner status, and prior HPV awareness. The relative indirect effects remained stable and significant across both models, confirming the robustness of the mediation mechanism independent of potential confounders.

Abbreviations: HA, Health Anxiety; eHL, eHealth Literacy; CI, Confidence Interval.
